# Combined *In Silico, In Vivo,* and *In Vitro* Studies Shed Insights into the Acute Inflammatory Response in Middle-Aged Mice

**DOI:** 10.1371/journal.pone.0067419

**Published:** 2013-07-02

**Authors:** Rami A. Namas, John Bartels, Rosemary Hoffman, Derek Barclay, Timothy R. Billiar, Ruben Zamora, Yoram Vodovotz

**Affiliations:** 1 Department of Surgery, University of Pittsburgh, Pittsburgh, Pennsylvania, United States of America; 2 Immunetrics, Inc., Pittsburgh, Pennsylvania, United States of America; 3 Department of Mathematics, University of Pittsburgh, Pittsburgh, Pennsylvania, United States of America; 4 Center for Inflammation and Regenerative Modeling, McGowan Institute for Regenerative Medicine, University of Pittsburgh, Pittsburgh, Pennsylvania, United States of America; New York University, United States of America

## Abstract

We combined *in silico*, *in vivo*, and *in vitro* studies to gain insights into age-dependent changes in acute inflammation in response to bacterial endotoxin (LPS). Time-course cytokine, chemokine, and NO_2_^−/^NO_3_^−^ data from “middle-aged” (6–8 months old) C57BL/6 mice were used to re-parameterize a mechanistic mathematical model of acute inflammation originally calibrated for “young” (2–3 months old) mice. These studies suggested that macrophages from middle-aged mice are more susceptible to cell death, as well as producing higher levels of pro-inflammatory cytokines, vs. macrophages from young mice. In support of the *in silico*-derived hypotheses, resident peritoneal cells from endotoxemic middle-aged mice exhibited reduced viability and produced elevated levels of TNF-α, IL-6, IL-10, and KC/CXCL1 as compared to cells from young mice. Our studies demonstrate the utility of a combined *in silico*, *in vivo*, and *in vitro* approach to the study of acute inflammation in shock states, and suggest hypotheses with regard to the changes in the cytokine milieu that accompany aging.

## Introduction

Inflammation ensues upon acute biological stress such as bacterial infection or tissue trauma [1]. In elderly individuals, the inflammatory response becomes radically altered, and is often accompanied by low grade, chronic, inflammation even in the absence of external stimulus [2]–[4]. Indeed, the linkage between inflammation and aging is close, prompting some authors to coin the term “inflamm-aging” for this complex process [4]. “Inflamm-aging” is associated with many functional alterations in both innate and adaptive immunity [5], [6]. Among the cellular components of the innate immune response, macrophages secrete a wide range of cytokines, chemokines and growth factors in response to pathogens, bacterial toxins, and host-derived damage-associated molecular pattern (DAMP) molecules [7], [8]. The effect of age on the production of these inflammatory mediators following macrophage activation by stimuli such as Gram-negative bacterial lipopolysaccharide (LPS) is not well defined [9]–[11].

To address the complexity of the acute inflammatory response *in vivo*, we have constructed mechanistic mathematical models of increasing complexity, using ordinary differential equations (ODE), which encompass the dynamics of relevant cells and cytokines as well as aspects of physiology and global tissue damage/dysfunction [12]–[15]. These computational models are capable of quantitative predictions, since they are calibrated to circulating levels of inflammatory mediators in mice [12], [16], rats [17], and swine [18]. These models’ ability to predict mortality is based on the incorporation of a global tissue damage/dysfunction variable, which is both a marker of the adverse effects of inflammation as well as a driver of further inflammation.

To address the effect of a chronic inflammatory perturbation on inflammation *in vivo*, we have developed a methodology that utilizes a combination of the mathematical model, automated data-fitting algorithms, and both *in vivo* and *in vitro* data. We previously utilized this approach to examine the controversial role of LPS or bacterial translocation in trauma/hemorrhage-induced inflammation [16]. Herein, we sought to use a similar approach to gain insight into the alterations characteristic of inflammation in aged mice. We hypothesized that we could obtain insights into *cellular-level* changes characteristic of the aged inflammatory response by examining the *systemic* responses of “middle-aged” mice, re-parameterizing our mathematical model for the levels of circulating inflammatory analytes in these mice, and deriving insights into the cellular and intracellular changes that could lead to these systemic alterations.

## Materials and Methods

### Ethics Statement

This study was performed in strict accordance with animal use protocols following approval of the University of Pittsburgh Institutional Animal Care and Use Committee (IACUC, protocol number 13021433). These studies involved experimentally-induced acute inflammation (endotoxemia; see below). Clinical signs associated with this experimental paradigm include decreased food/water intake, lethargy, anorexia, piloerection, and weight loss. These pathophysiological changes mandated the classification of this study as Category E of Pain/Distress. Euthanasia via approved methods at a maximum of 90 min, or immediately upon observation of the above clinical signs, was the primary means of mitigating this degree of pain and distress.

Analgesics (opioids, steroids and non-steroidal anti-inflammatory drugs) have profound effect on the cardiorespiratory system and on the inflammatory response [19]. These effects include blockade of cytokine response and acceleration of the progression to shock [20]. In rodent models of sepsis, trauma-hemorrhagic shock and hepatic ischemia/reperfusion, these drugs are also known to significantly alter the inflammatory response and the degree of organ damage [21]. Because the study proposed in this protocol aim to unravel the mechanisms of inflammation-induced organ injury through the generation of mathematical models, administration of these drugs was contra-indicated, for they would alter the course of inflammation and injury.

### Experimental Endotoxemia and Culture of Resident Peritoneal Cells

Male C57BL/6 mice were purchased from Charles Rivers (Wilmington, MA) and were acclimatized in specific pathogen-free conditions with 12-h light/dark facilities and received food and water *ad libitum*. Plasma levels of inflammatory mediators from young mice (n = 4, 8–12 weeks old, weighing 20–25 g) were used for the initial calibration of the mathematical model, as described elsewhere [12]. Plasma levels of inflammatory mediators from “Middle-aged” mice (n = 4, 6–8 months old, weighing 25–35 g) were used to re-parameterize the mechanistic mathematical model of acute inflammation that was originally calibrated for young mice (see ***Mathematical model of inflammation***). In these initial studies, a limited number of plasma analytes (TNF-α, IL-6, IL-10, and NO_2_^−/^NO_3_^−^; see ***Cytokine and NO_2_^−/^NO_3_*** ^−^ ***analysis***) were assessed. These initial re-calibration studies led specific predictions regarding biological properties of young vs. middle-aged macrophages (see ***Mathematical model of inflammation***).

To validate these predictions *in vitro*, additional mice (n = 16 for either young or middle-aged) were treated with sterile saline or 3 mg/kg LPS (*Escherichia coli* serotype O111:B4, catalog # L2630; Sigma-Aldrich, St. Louis, MO) intraperitoneally for 90 min. The animals were then euthanized by CO_2_ asphyxiation followed by cervical dislocation. Blood samples were collected via cardiac puncture and stored at −80°C until analysis for cytokine and NO_2_^−/^NO_3_^−^.

To obtain resident peritoneal cells, the peritoneal cavity was then flushed with cold saline (10 ml) via a large bore needle (18-gauge), avoiding traumatic perforation of the bowel. The abdomen was gently manipulated and the peritoneal fluid was aspirated back using a 26-gauge needle. Peritoneal cells were collected, washed and suspended in previously prepared DMEM (Dulbecco’s Modified Eagle’s Medium, Lonza, Walkersville, MD) cell culture media containing 10% heat-inactivated FCS, 1 mM glutamine, 50 U/ml penicillin and 50 µg/ml streptomycin.

Peritoneal cells (at a density of 2×10^5^ cells per well) from both saline and LPS treated mice were plated on eight-well Lab Tek™ Chamber Slide™ tissue culture plates (Nalge Nunc International, Rochester, NY) and allowed to attach in a humidified atmosphere (5% CO_2_) for 2 h [22]. After adherence, the culture media was replaced to remove the non-adherent cells and cell monolayers (predominantly to be macrophages) were further incubated for 90 min. At the end of the experiment, the supernatants were collected from each well and stored at −80°C until analysis. To determine peritoneal cell count and viability, 10 µL of peritoneal fluid were mixed with an equivalent volume of 0.1% trypan blue pipetted into Countess® chamber slide, and analyzed using a Countess® Automated Cell Counter (Life Technologies, Grand Island, NY).

### Qualitative Assessment of Cultured Peritoneal Macrophages

At the end of the cell culture experiments the supernatants were collected and the purity and overall viability of the resident peritoneal macrophages were assessed. To do so, the wells were washed with PBS and fixed with 70% ethanol. Eight random wells from two chamber slides were selected from each group (young and middle-aged mice) and macrophages were identified in each well by characteristic morphology under light microscopy as [23].

### Cytokine and NO_2_^−/^NO_3_^−^ Analysis

For the initial calibration of the mathematical model, plasma samples from young mice (n = 4) treated with LPS *in vivo* were collected at 0, 60 min, 90 min, 4 h, 12 h and 24 h and assayed for IL-6, TNF-α, IL-10, and NO_2_^−/^NO_3_^−^. For the validation cohort of mice, plasma samples and cell culture supernatants (n = 16 per group for young and middle-aged mice, respectively) were assayed for 20 cytokines using a multiplex bead immunoassay system (Luminex™, Millipore, Billerica, MA). The cytokine assays included granulocyte-macrophage colony stimulating factor (GM-CSF), keratinocyte-derived chemokine (KC; CINC-1; CXCL1), interferon-γ (IFN-γ), IFN-γ-inducible protein-10 (IP-10; CXCL10), interleukin- (IL)-1α, IL-1β, IL-2, IL-4, IL-5, IL-6, IL-10, IL-12 p40, IL-12p70, IL-13, IL-17, monocyte chemotactic protein-1 (MCP-1; CCL2), monokine induced by gamma interferon (MIG; CXCL9), macrophage inflammatory protein-1α (MIP-1α), *vascular endothelial growth factor* (VEGF) and tumor necrosis factor-α (TNF-α). Plasma and supernatant NO_2_^−/^NO_3_^−^ were measured by the nitrate reductase method using a commercially available kit (Cayman Chemical, Ann Arbor, MI).

### Statistical Analysis

All data are expressed as mean ± SEM. Statistical analysis was performed by Student’s t-test or Two-Way ANOVA followed by Tukey *post hoc* or Holm-Sidak test as indicated using SigmaPlot (Systat Software, San Jose, CA), with *P*<0.05 considered to be significant.

### Mathematical Model of Inflammation

The equations and specifications of the “young mouse-specific” mathematical model of inflammation have been described elsewhere [12]. Briefly, we constructed a mathematical model of acute inflammation that incorporates key cellular and molecular components of the acute inflammatory response. The mathematical model consists of a system of 17 nonlinear ordinary differential equations that describe the time course of these components. Included in the model equations are two systemic variables that represent mean arterial blood pressure and global tissue dysfunction and damage. “Global tissue damage/dysfunction” describes the overall health of the organism, since the hallmark of all physiological derangements accompanying sepsis and hemorrhagic shock is the eventual, sequential failure of multiple organs. Given the complexity of simulating individual organs, we approximated this process by treating it as a gradual, ongoing process occurring in the whole body and driven by inflammation. Thus, elevated and unrecoverable tissue damage/dysfunction served as a surrogate for death, while damage/dysfunction that tended to return to baseline over time was a proxy for survival.

Each equation was constructed from known interactions among model components as documented in the existing scientific literature. The model and parameters were specified in three stages. In the preliminary stage, the model was constructed so it could reproduce qualitatively several different scenarios reported in the literature. In this stage, experimentally determined values of parameters such as cytokine half-lives were used when available. In the second stage, the model was matched to our experimental data by adjusting some of the parameters using our qualitative understanding of the biological mechanisms together with the dynamics of the model, to attain desired time course shapes. In the third stage, the parameters were estimated by fitting the model to the experimental data and using a stochastic gradient descent algorithm that was developed by Immunetrics, Inc. (Pittsburgh, PA) [12].

### Adaptation of “Young-specific” Mathematical Model to Account for Inflammation in Aging Mice

**Figure 1:**
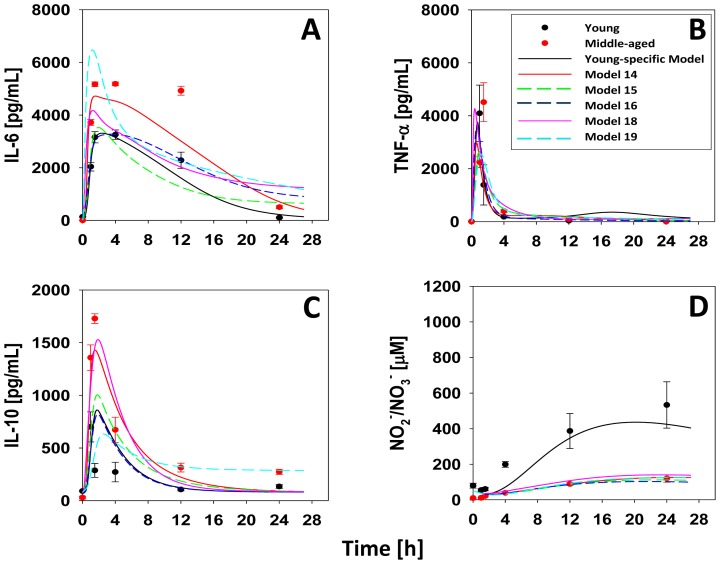
Data and model output for plasma cytokines and NO_2_^−/^NO_3_^−^ in young and middle-aged mice subjected to LPS. Young (C57BL/6; n = 4 animals per time point) and middle-aged mice (n = 4 per time point) were injected with saline or 3 mg/kg LPS as described in *Materials and Methods*. Plasma concentration as a function of time and age: (**A**) IL-6, (**B**) TNF-α, (**C**) IL-10 and (**D**) NO_2_^−/^NO_3_^−^. Symbols represent the mean ± SEM (*P*<0.05, analyzed by Two-way ANOVA followed by Holm-Sidak test for plasma levels of IL-6 and IL-10). For young mice, the line indicates the output of the baseline model of acute inflammation. For middle-aged mice, model re-calibration was carried out as described in the *Materials and Methods*, yielding five models (colored lines). “Best Model” indicates the model giving the best overall fit to the data.

To recalibrate our mathematical model for data obtained in “middle-aged” mice we sought to find a minimal number of parameter changes that would allow our original model to reproduce accurately empirically observed time courses in aging-mice. To prevent the optimizing algorithm from exploiting the indirect action of unmeasured analytes, we attempted to confine our search to those parameters that appear directly in the equations of our measured analytes (IL-6, TNF-α, IL-10, and NO_2_^−/^NO_3_^−^). Our procedure was to impose a limit of N changes, run an optimizer bounded by this change limit, and evaluate the fit. We evaluated these fits with respect to quantitative error totals as well as general qualitative behavior. After evaluation, we increased the number of parameters to be optimized and repeated the process iteratively until sufficient fit quality was achieved. Each optimization pass was allowed to choose randomly which N of the candidate parameters to change. We performed numerous fits for each N to account for the stochastic nature of our gradient descent search. In order to obtain good fits with fewer than 10 changes, we found it necessary to add the parameters of the (unmeasured) activated macrophages (*MA*), activated neutrophils (*NA*), and iNOS equations to our initial set of parameters to be fit [12]. When these parameters were included in the search, we found that excellent fits could be obtained with fewer than eight parameter changes. We also observed that eliminating any one of these changes seems to result in a worse fit by both quantitative and qualitative measures. This procedure yielded five parameter sets with good fits to the data in middle-aged mice, with essentially equal measures of error with respect to experimental data (data not shown). The models exhibited similar behavior with regard to model output and predictions presented herein, with various “tradeoffs” relative to a hypothetical perfect fit. Each parameter set was based on similar, but not identical, changes to various constants (Table 1). We show these models and indicate the one judged as the best overall fit to the data by investigator consensus (necessary given the overall similarity of the quantitative measures used to distinguish among the models). Nonetheless, the model output and predictions of all models are shown (Fig. 1, lines).

**Table 1:** Characteristics of middle-aged specific mathematical models of inflammation.

*Model*	Param.	*h10cp*		*hn10*	*kmd*			*kmpe*			*kn*		*knpe*	xcp6	
**Best**	*Param. Def.*	Influence of TNF onIL-10 (Hill coefficient)		Influence of IL-10 on leukocyte activation (Hill coefficient)	Rate of activation of macrophages due to damage			Activation of macrophages by LPS			Death rate of neutrophils		Activation of neutrophils by LPS	Saturation constant for IL-6′s influence on TNF	
	*Change in Middle-* *aged vs. Young*	8-fold lower		5-fold lower	3-fold lower			9-fold higher			16-fold higher		5-fold higher	11-fold higher	
***Model 15***	***Param.***	***h6cp***	***k10***		***k10m0***		***kn***			***s6***		***xcp6***			
	*Param. Def.*	Influence of TNF onIL-10 (Hill coefficient)	Regulates levels of IL-10 by modulating production/decay rates		Rate of IL-10 production in response to other cytokines	Death rate of neutrophils			Propensity to produce and secrete IL-6 at rest			Saturation constant for IL-6′s influence on TNF			
	*Change in Middle-* *aged vs. Young*	3-fold lower		7-fold higher	84-fold higher			4-fold higher			36-fold higher		66-fold higher		
***Model 16***	***Param.***	***k6no***		***Kn***	***knpe***			***s6***			***xcp6***		***xm6***		
	*Param. Def.*	Influence of NO_2_^−/^NO_3_^−^ on IL-6		Death rate of neutrophils	Activation ofneutrophils by LPS			Propensity to produce and secrete IL-6 at rest			Saturation constant for IL-6′s influence on TNF		Saturation constant for IL-6′s influence on macrophage activation		
	*Change in Middle-* *aged vs. Young*	26-fold higher		19-fold higher	6-fold higher			46-fold higher			50-fold higher		3-fold higher		
***Model 18***	***Param.***	***hcp6***		***hcp10***	***kinosn***			***knpe***			***s6***		***x6cp***	***xmpe***	
	*Param. Def.*	Influence of IL-6 onTNF (Hill coefficient)		Influence of IL-10 onTNF (Hill coefficient)	Influence ofneutrophils on iNOS			Activation ofneutrophils by LPS			Propensity to produce and secrete IL-6 at rest		Saturation constant for TNF’s influence on IL-6	Saturation constant for influence of LPS on macrophage activation	
	*Change in Middle-* *aged^/^vs. Young*	1.4-fold lower		41-fold lower	6-fold lower			28-fold higher			62-fold higher		73-fold higher	7-fold lower	
***Model 19***	***Param.***	***k6n***		***kn***	***s10***			***xcp6***			***xmd***				
	*Param. Def.*	Influence ofneutrophils on IL-6		Death rate of neutrophils	Propensity to produce and secrete IL-10at rest			Saturation constant for IL-6′s influence on TNF			Saturation constant for influence of damage on macrophage activation				
	*Change in Middle-* *aged vs. Young*	52-fold higher		8-fold higher	4-fold higher			30-fold higher			7-fold higher				

Model parameters (constants) changed in the middle-aged specific models are indicated, along with their biological significance. Param.: parameter. Def.: definition.

## Results

### Age-associated Changes in Inflammatory Mediators of Endotoxemic Mice

**Figure 2:**
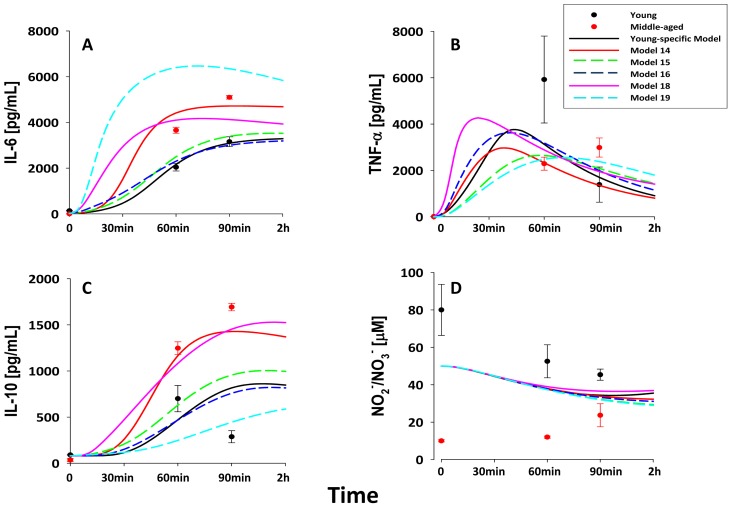
Difference in plasma cytokines and NO_2_^−/^NO_3_^−^ in young and middle-aged mice subjected to LPS. Young (C57BL/6; n = 3–8 animals per time point) and middle-aged mice (n = 4 per time point) were injected with saline or 3 mg/kg LPS as described in *Materials and Methods*. Middle-aged mice had significantly higher levels of IL-6 (**A**), TNF-α (**B**), and IL-10(**C**) at 60 and 90 min when compared to young mice post-LPS treatment (*P*<0.05, analyzed by Two-way ANOVA).

Prior studies had suggested that older mice exhibit dysregulated acute inflammation [24], [25]. We confirmed this finding initially by measuring plasma levels of IL-6 (Fig. 1A), TNF-α (Fig. 1B), IL-10 (Fig. 1C) and NO_2_^−/^NO_3_^−^ (Fig. 1D) in “young” (2–3 month old) and “middle-aged” (6–8 month old) C57BL/6 mice subjected to bolus intraperitoneal injection of 3 mg/kg LPS. Based on Two-Way ANOVA, plasma levels of IL-6 and IL-10 were statistically significantly higher (P<0.05) in middle-aged mice when compared to similarly treated young mice, whereas NO_2_^−/^NO_3_^−^ was statistically significantly higher (P<0.05) in young mice. In addition, we discerned significant (P<0.05) age-time interactions in the dynamics of IL-6 (at 60 min, 90 min, 4 h and 12 h), TNF-α (at 60 and 90 min), IL-10 (at 60 min, 90 min, 4 h and 12 h), and NO_2_^−/^NO_3_^−^ (at 4 h, 12 h and 24 h) (Fig. 2, symbols), when comparing young vs. middle-aged mice post-LPS treatment.

### Mathematical Modeling of Age-dependent Changes in Circulating Inflammatory Mediators

We next hypothesized that mathematical modeling could help us gain insights into the some of these complex changes in circulating inflammatory mediators. We have previously developed a mechanistic mathematical model in which dynamics of leukocytes and their products affect the whole-organism inflammatory response assessed as circulating levels of mediators, that we calibrated using data from young C57BL/6 mice [12], [16], [26], [27] (see *Materials and Methods*). Using this base mathematical model, we have previously developed a methodology by which to gain insight into the complex changes in the acute inflammatory response of gene-deficient animals (see *Materials and Methods*), which we utilized to study the complex alterations in the acute inflammatory response of CD14-deficient mice [16]. This methodology involves measuring the levels of key circulating inflammatory mediators (IL-6, TNF-α, IL-10, and NO_2_^−/^NO_3_^−^); re-parameterizing the base mathematical model for these new data by generating an ensemble of models with roughly equivalent fit to the data, as assessed by an error function; and finally, determining the consensus set of parameters that were altered in the automated fitting process in order to fit these new data. Since these parameters can be mapped to cellular- and molecular-level biological processes (e.g. the degree to which LPS will induce macrophages to produce TNF-α), this re-parameterization process allows us to generate hypotheses regarding the underlying differences in the inflammatory responses of the test animals (in the current study, middle-aged C57BL/6 mice) vs. the control animals (young C57BL/6 mice) based solely on systemic inflammatory mediator data.

Accordingly, we carried out the process described above (see *Materials and Methods*). The resulting ensemble of five mathematical models that fit the systemic IL-6, TNF-α, IL-10, and NO_2_^−/^NO_3_^−^ data in middle-aged mice suggested that, at the cellular level, aged leukocytes produce higher levels of inflammatory cytokines, particularly IL-6, IL-10 and to a lesser degree TNF-α at baseline and in response to LPS. These models also predicted that leukocytes from middle-aged mice undergo increased death as compared to leukocytes from young mice (Table 1 and Fig. 1, lines).

### *In vitro* Validation of *in silico* Predictions

We next sought to validate these mathematical model predictions by *in vitro* studies using resident macrophages harvested from peritoneal cavities of young and middle aged mice that received either intra-peritoneal saline or LPS. We first confirmed that treatment with LPS for 90 min would result in a broad-based induction of the inflammatory response *in vivo*. Accordingly, we expanded our analysis to additional animals and additional circulating inflammatory mediators (20 cytokines/chemokines as well as NO_2_^−/^NO_3_^−^) (Table 2). In support of our initial findings, middle-aged mice that were treated with LPS *in vivo* had statistically significantly higher plasma concentrations of IL-6, TNF-α, IL-10, MIG/CXCL9, MCP-1/CCL2, IL-1α, IL-17, and IL-12p40 when compared to LPS treated young mice. However, saline-treated (baseline) middle-aged mice had higher circulating levels of GM-CSF, IL-1β, IL-2, IL-4, IL-12p40, and VEGF when compared to saline-treated young mice. Moreover, saline-treated young mice exhibited statistically significantly higher circulating levels of NO_2_^−/^NO_3_^−^ when compared to saline treated middle-aged mice (Table 2). Circulating plasma levels of IFN-γ, IL-12p70, IL-13, IL-17 and MIG were below the detection limit in both young and middle-aged mice after either saline or LPS treatment.

**Table 2:** Statistically significant plasma inflammatory mediators in both young and middle-aged mice after I.P saline or LPS treatment.

Treatment	Inflammatory Mediators	Young (mean ± SEM)	Middle-aged (mean ± SEM)	*P* value (Student’s t-test)
Saline	GM-CSF	0	20.2±3.5	*P*<0.001
	IL-1β	0	21.2±2.9	*P*<0.001
	IL-2	2.7±0.2	4.8±0.2	*P*<0.001
	IL-4	2.8±0.2	5.5±0.4	*P*<0.001
	IL-12p40	0	28.9±2.8	*P*<0.001
	VEGF	1.2±0.2	2.8±0.3	*P*<0.001
	NO_2_^−/^NO_3_^−^	36.9±1.9	22.1±1.1	*P*<0.001
LPS	IL-6	14589.7±1583.9	33445.08±7281.9	*P* = 0.004
	TNF-α	93.6±18.1	332.6±63.7	*P*<0.001
	IL-10	251.9±40.3	458.9±67.2	*P* = 0.016
	MIG/CXCL9	100.9±17.5	4099.4±690.6	*P*<0.001
	MCP-1	18869.1±3153.4	34142.9±4515.6	*P* = 0.012
	IL-1α	48.1±6	124.5±0.4	*P* = 0.002
	IL-17	0	5.5±0.9	*P*<0.001
	IL-12p40	104.1±14.3	183.2±21.3	*P* = 0.006
	NO_2_^−/^NO_3_^−^	54.7±4.3	34.6±2.3	*P*<0.001
	GM-CSF	59.4±5.9	35.4±4.8	*P* = 0.005

Young and middle-aged mice (n = 16 each) were treated with either saline or LPS *in vivo* for 90 min., and circulating inflammatory mediators were assessed as described in *Materials and Methods*.

**Figure 3:**
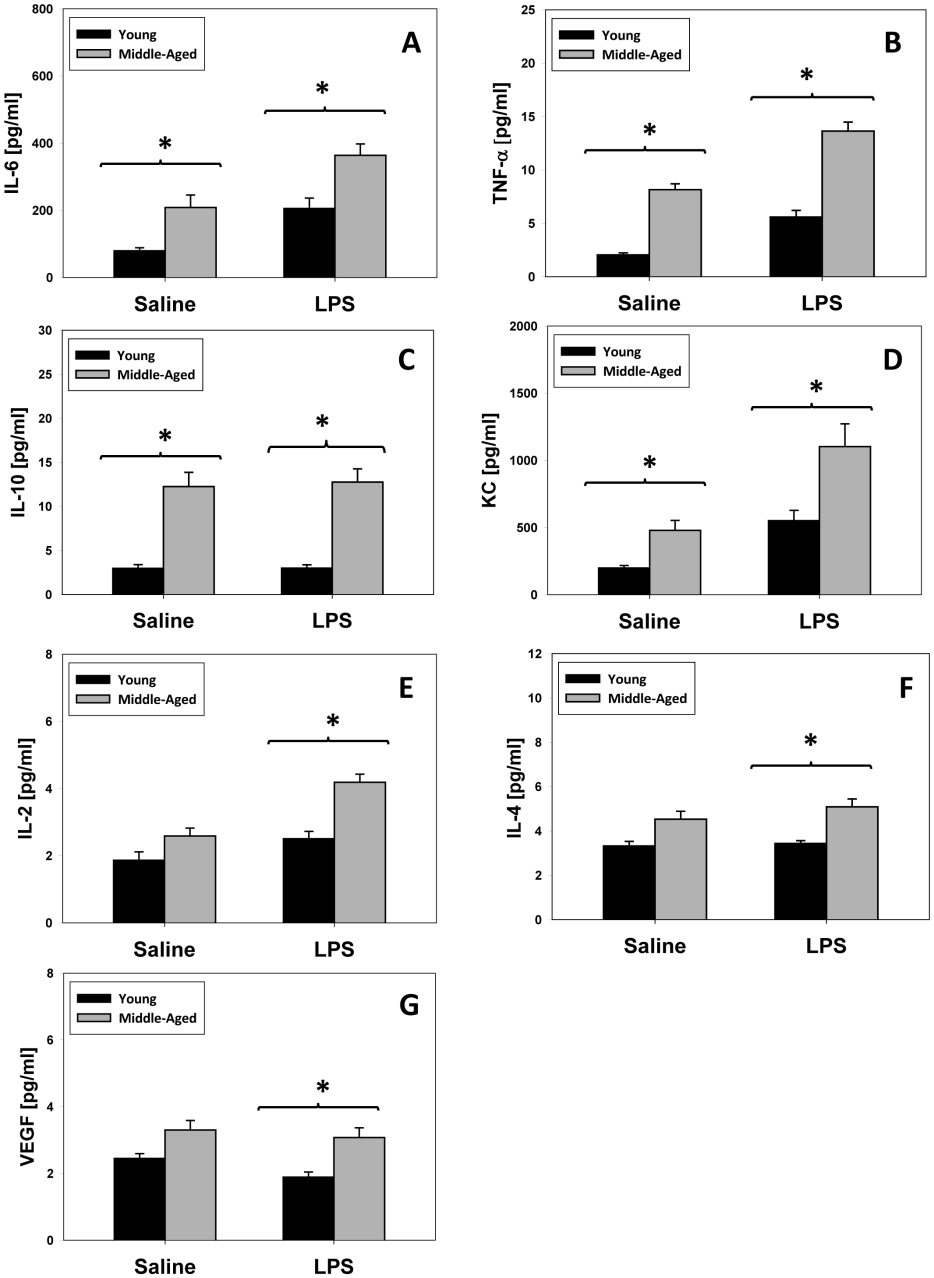
*In vitro* cytokine concentrations after *in vivo* treatment with either saline or LPS in young and middle aged mice. Young (n = 16) and middle-aged mice (n = 16) were injected with saline or 3 m/kg LPS and peritoneal macrophages were harvested as described in *Materials and Methods*. Middle-aged mice had significantly higher levels (*P<0.001 vs. young mice) of IL-6 (**A**), TNF-α (**B**), IL-10 (**C**) and KC (**D**) after *in vivo* treatment with saline followed by *in vitro* treatment with culture media. IL-6 (**A**), TNF-α (**B**), IL-10 (**C**), KC (**D**), IL-2 (**E**), IL-4 (**F**) and VEGF (**G**) concentrations were significantly higher (*P<0.001 vs. young mice) after *in vivo* LPS treatment followed by *in vitro* treatment with culture media.

One key prediction of our re-parameterized mathematical model of inflammation was that leukocytes from middle-aged mice would exhibit a higher production of inflammatory cytokines. Resident macrophages from young and middle-aged mice were obtained by peritoneal lavage and the supernatants of adherent cells were assayed for the same 21 inflammatory mediators described in Table 2. In support of the *in silico* predictions, macrophages from saline-treated middle-aged mice produced elevated levels of IL-6 (Fig. 3A), TNF-α (Fig. 3B), IL-10 (Fig. 3C) and KC (Fig. 3D) when compared with similarly treated young mice. However, macrophages from middle-aged mice subjected to 3 mg/kg LPS *in vivo* secreted statistically significantly elevated levels of IL-6 (Fig. 3A), TNF-α (Fig. 3B), IL-10 (Fig. 3C), KC (Fig. 3D), IL-2 (Fig. 3E), IL-4 (Fig. 3F) and VEGF (Fig. 3G) as compared to similarly-treated macrophages from young mice. We note that IL-2, IL-4, and VEGF are not included in the mathematical model of inflammation [12].

Given the mathematically-predicted, age-dependent difference in baseline and *in vivo* LPS-induced production of inflammatory mediators, we next sought to determine if the actual degree of LPS-induced stimulation of these mediators differed between young and middle-aged mice. Accordingly, we calculated the fold change difference among those mediators determined to be statistically significantly different between young and middle-aged mice. This fold change difference was calculated as follows:
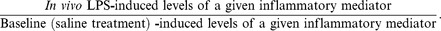



Figure S1 shows fold change difference between young and middle-aged mice. This analysis showed a statistically significant (*P* = 0.004) higher fold change difference for TNF-α in young mice when compared to middle-aged mice, whereas there was no statistical differences in the other inflammatory mediators.

Only one of the five middle aged-specific mathematical models invoked a relatively minor effect on *kinosn*, a parameter governing the influence of neutrophils on iNOS. Largely in agreement with the predictions of the mathematical model, there were no statistically significant differences in NO_2_^−/^NO_3_^−^ levels in macrophages isolated from young or middle-aged mice that received either saline or LPS *in vivo.*

**Figure 4:**
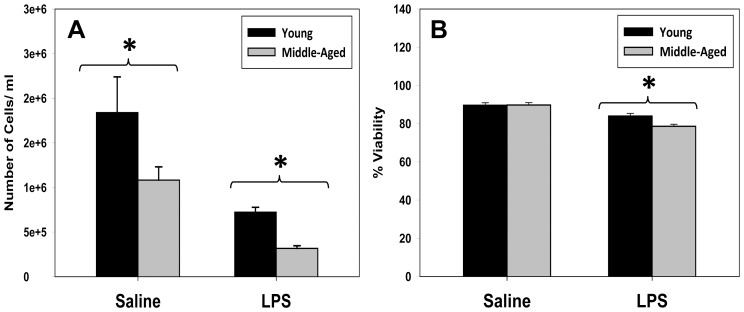
Total peritoneal cell count and cell viability. (**A**): Middle-aged mice that received *in vivo* LPS had significantly lower (*P<0.001) cell count when compared to similarly treated young mice. (**B**): LPS-treated middle-aged mice had significantly lower (*P = 0.008) cell viability when compared to LPS treated young mice.

Another key prediction derived from the re-parameterization of our mathematical model of inflammation was that leukocytes from middle-aged mice would exhibit a shorter lifespan as compared to leukocytes from young mice. Fig. 4 shows the total peritoneal cell count (Fig. 4A) and cell viability (Fig. 4B) in young and middle-aged mice after treatment with either saline or 3 mg/kg LPS *in vivo*. In support of the *in silico* prediction, peritoneal cells from young mice that were treated with saline or LPS *in vivo* had statistically significantly higher cell counts when compared to saline treated middle-aged mice (Fig. 4A). Peritoneal cells from LPS-treated, but not saline-treated middle-aged mice exhibited significantly lower cell viability when compared to cells from LPS-treated young mice (Fig. 4B).

## Discussion

Aging and the diseases that stem from it are consuming an ever-larger portion of healthcare expenditures [28]. Recent genome-wide association studies suggest that inflammation and cellular senescence are common mechanisms in aging-associated diseases [29]. Such studies raise the hypothesis that dysregulated inflammation is at least part of the cause of aging-related diseases [2], [3], [30], [31], which has been supported by studies demonstrating inflammation-counterbalancing mechanisms in centenarians [32]. It has also been hypothesized that an age-dependent increase in pro-inflammatory tendency was favored in an environment in which infection was prevalent, but that elevated inflammation is predominantly detrimental in a modern world in which infection is much less prevalent [33]. Importantly, these changes in inflammation can be observed by middle age in both humans and experimental animals, and are not merely a hallmark of the very aged [34]–[36].

Monocytes and macrophages play central roles in the initiation and resolution of inflammation in response to various insults [37], including LPS-induced endotoxic shock [8]. Monocytes and macrophages originate from a common myeloid progenitor cell in the bone marrow and, under normal circumstances; monocytes circulate in the bloodstream for a very short time before undergoing spontaneous apoptosis [37], [38]. In response to differentiation factors, monocytes escape their apoptotic fate by differentiating into tissue-resident macrophages [39]. Studies of age-related differences in macrophage production of pro-inflammatory and anti-inflammatory cytokines in response to acute stimulation *in vitro* have yielded conflicting results, highlighting the complexity of inflammation in the aging process. Indeed, some groups have reported a decrease in levels of IL-1β [40], IL-6 [41], and TNF-α [42], while increases in levels of IL-1β, IL-6, and TNF-α [43] have been reported by others.

Remarkable advances have been made in revealing molecular mechanism underlying the acute inflammatory response, and yet the complexity of this process – with its redundancy, positive and negative feedback loops, and emergent system behavior that cannot be readily discerned from a reductionist analysis of individual pathways – makes inflammation the prototypical case study for the application of systems and computational biology [44], [45]. These approaches, which include various “omics” methods as well as computational modeling, are being utilized to address the complexity of inflammation in the aging process [46].

Accordingly, we hypothesized that we could gain cellular-level insights into the altered inflammatory response that accompanies aging using a mechanistic computational model that simulates whole-animal inflammation as a consequence of inflammatory cell activation by LPS [12], [16], [26], [27]. We utilized mouse endotoxic shock as a simplified paradigm of acute inflammation driven in large part by monocyte/macrophages [8]. In essence, the variables and parameters in our mathematical model represent different aspects of cellular and sub-cellular biology of inflammation (e.g. growth and decay rates of cells and cytokines), and is predicated on the systemic spillover of inflammatory mediators into the circulation. This latter feature – the connection between local and systemic inflammation – has allowed us to calibrate this model using data on circulating inflammatory mediators. We have previously utilized this mathematical model to show commonality among the responses to LPS, surgical trauma, and trauma/hemorrhage in young C57BL/6 mice [12]. Using this mathematical model, we have also gained insights into the characteristics of systemic inflammation in young CD14-deficient mice vs. young C57BL/6 controls, including the lack of a role for endogenous LPS in trauma/hemorrhage-induced inflammation [16].

We reasoned that we could gain mechanistic insights into the role of LPS in the inflammatory response by obtaining data on circulating cytokines and NO_2_^−/^NO_3_^−^ in middle-aged mice, re-calibrating our model, and determining which parameters were changed relative to young mice. In initial studies, middle-aged mice subjected to 3 mg/kg LPS exhibited higher circulating levels of IL-6 and IL-10, and blunted NO_2_^−/^NO_3_^−^ when compared to young mice, and in subsequent studies we verified that a broader set of mediators was likewise altered in middle-aged mice. We then re-calibrated the baseline (young C57BL/6 mouse-specific) mathematical model of inflammation using automated algorithms to yield an ensemble of “aging-specific” models. These models were interrogated as to changes in various parameters (corresponding to biological mechanisms) compared to the “young-specific” model. The ensemble of five mathematical models that best fit these data suggested that aged leukocytes will produce elevated *baseline* levels of IL-6 (and to a lesser degree, TNF-α), elevated LPS-induced production of pro-inflammatory mediators and undergo increased cell death as compared to young leukocytes, in agreement with the literature [36], [47].

To validate key *in silico* predictions about cellular-level changes in acute inflammation that occur with aging, predictions which were based on *in vivo* data (levels of circulating inflammatory mediators), we carried out *in vitro* time-course studies on peritoneal macrophages from C57BL/6 young and middle-aged mice. To assess the universality of these predictions, and to gain further insights into changes in acute inflammation that are characteristic of aging, we surveyed a larger number of inflammatory mediators. These *in vitro* results largely validated the mathematical model prediction, as observed by elevated levels of IL-6, TNF-α, IL-10 and KC in resident peritoneal cells from middle-aged mice as compared to cells from similarly-treated young mice. Though aging can lead to reduced macrophage NO production [48], our algorithms suggested at best a minor role for reduced expression of iNOS by neutrophils, and this prediction was supported by the lack of statistically significant differences in the production of NO_2_^−/^NO_3_^−^ between young and middle-aged peritoneal macrophages. In addition, the statistically significant fold change difference in TNF-α production suggests that macrophages in young mice have greater responsiveness to LPS *in vivo,* with regard to this key inflammatory cytokine, when compared to similarly treated macrophages in middle-aged mice. Importantly, other inflammatory mediators that differ significantly between young and middle-aged mice are induced by LPS to roughly the same extent. Another key *in silico* prediction was that the lifespans of leukocytes derived from older mice would be shorter, a prediction supported by *in vitro* cell qualitative assessment and cell viability as well.

Our experimental studies, like our mathematical model, were focused on cellular-level phenomena that drive systemic changes in inflammatory mediators. In the present study, we therefore sought to stimulate animals *in vivo* and then examine the responses *in vitro* with the least further perturbation, in order to verify predictions derived from the mathematical model. In separate studies we also treated cultured macrophages with LPS *in vitro* (data not shown). We observed a roughly similar increase in the levels of inflammatory mediators when we added LPS *in vitro* to macrophages derived from mice that were previously treated *in vivo* with saline (data not shown). Interestingly, *in vitro* LPS treatment of macrophages derived from either young or middle-aged mice previously treated *in vivo* with LPS resulted in *decrease* in levels of inflammatory mediators, suggesting that the phenomenon of LPS tolerance [49], [50] occurs to an approximately equal degree in young vs. middle-aged mice (data not shown).

We recognize that there are several limitations to the current study. We note that there is discrepancy in the literature regarding the definition of “middle-aged” mice. While some authors report “middle-age” as early as 8 month old [51], [52], others have considered 10–12 month as the appropriate range [53], [54]. Yet other investigators have gone beyond these age ranges and used 17–18 month mice to refer to “middle-age” [55], [56]. In our study, we used 6–8 month old mice to characterize the inflammatory response associated with the upper age range for the mature adult group, which is typically approximately *6 months* [*57*] *. We suggest that our reported changes in the acute inflammatory response might best be seen as characteristic of the continuum of aging.* Another limitation of our study lies with the cell type being studied. Like all mathematical models, our model is, by necessity, an abstraction of the full scope of the biology of acute inflammation. Thus, variables such as “macrophages” may represent multiple inflammatory cell types (e.g. dendritic cells). Also important is the fact that our mathematical model is based on a single compartment which obeys the mean-field approximation, i.e. the whole organism is abstracted as a “well-mixed bag”; hence, peritoneal inflammatory cells, chief among the being peritoneal macrophages, are the best analog of the “macrophage” variable in our model. Another limitation of our study is that while the mathematical model predicted an increase in cell death of leukocytes in middle-aged mice, it did not infer mechanisms by which cell death ensue in aged leukocytes. In the current study, Trypan blue stain was used to give a rough estimate of the viability of cultured macrophages and to validate the mathematical model prediction. Future studies will be aimed at understanding the mechanism of reduced macrophage viability, in concert with mathematical models of increased detail (e.g. including mechanistic details based on our prior work on modeling apoptosis [58] and the role of NO in modulating this form of programmed cell death [59]. In addition, we also note that macrophage quantification by morphology alone would yield rough estimation of the total macrophage count and that techniques such as FACS and macrophage-specific markers would be more accurate in macrophage quantification.

In conclusion, this study supports the concept of acute inflammation in older mice as a complex, dysregulated state in which imbalances in the cytokine network may lead to inappropriate, inadequate, or harmful responses. Moreover, our results demonstrate the utility of a combined *in silico*, *in vivo*, and *in vitro* approach to the study of acute inflammation.

## Supporting Information

Figure S1 Fold change difference in inflammatory mediators between young and middle-aged mice. Young mice had a statistically significant (*P* = 0.004) higher fold change in TNF-α when compared to middle-aged mice (panel B), whereas no statistical differences were observed in the other inflammatory mediators.
(TIF)
