# Supplementary figures and images for: Combined In Silico, In Vivo, and In Vitro Studies Shed Insights into the Acute Inflammatory Response in Middle-Aged Mice
Source: PLoS One. 2013 Jul 2;8(7):e67419. doi: 10.1371/journal.pone.0067419 (PMC3699569; doi:10.1371/journal.pone.0067419)

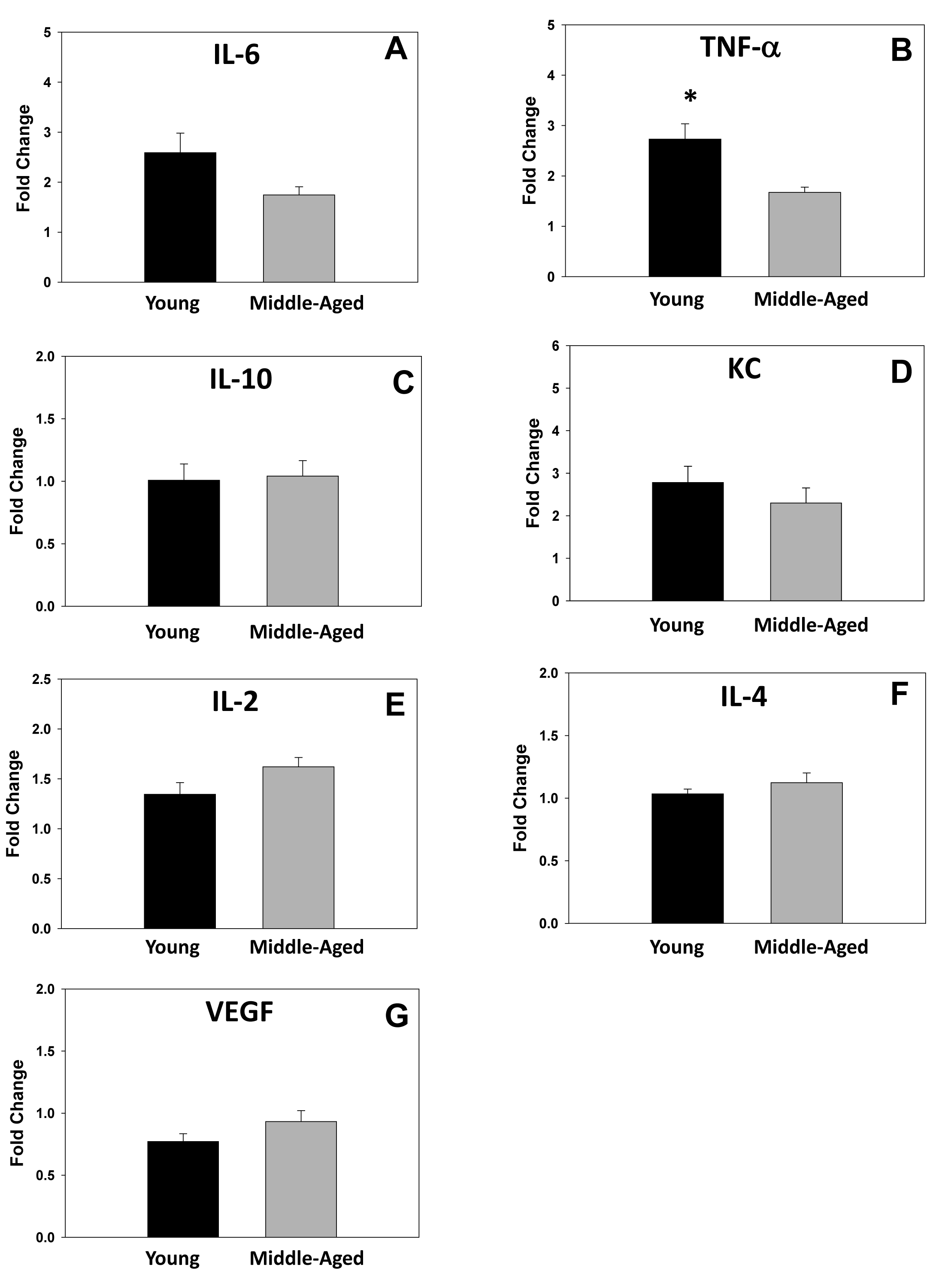

Supplement: Figure S1 — Fold change difference in inflammatory mediators between young and middle-aged mice. Young mice had a statistically significant (P = 0.004) higher fold change in TNF-α when compared to middle-aged mice (panel B), whereas no statistical differences were observed in the other inflammatory mediators. (TIF) [file pone.0067419.s001.tif]
